# How Severe is Severe Enough? Organ Failure Rather Than Anemia Severity as a Potential Threshold for Complement Inhibition in Pediatric Paroxysmal Cold Hemoglobinuria: A Case Report

**DOI:** 10.1155/crpe/8278542

**Published:** 2026-09-03

**Authors:** Colburn Yu, Zachary Rane, Elena Nedelcu, Alison Matsunaga

**Affiliations:** ^1^ Department of Pediatrics, UCSF Benioff Children’s Hospital Oakland, Oakland, California, USA; ^2^ Department of Pediatric Hematology Oncology, UCSF Benioff Children’s Hospital Oakland, Oakland, California, USA; ^3^ Department of Laboratory Medicine, UCSF Medical Center, San Francisco, California, USA, ucsfhealth.org

**Keywords:** autoimmune hemolytic anemia, case report, complement inhibition, Donath–Landsteiner antibody, eculizumab, paroxysmal cold hemoglobinuria

## Abstract

Paroxysmal cold hemoglobinuria (PCH) is an acquired intravascular hemolytic anemia of young children that is usually self‐limited. Two patients with PCH have previously been described in the medical literature to have received complement inhibition with eculizumab, an inhibitor of complement C5, and in both cases, the drug was given in the setting of organ failure. No criterion defines when the use of complement inhibition is warranted. We report a previously healthy 23‐month‐old boy with postviral PCH whose hemoglobin nadir of 2.7 g/dL, undetectable total hemolytic complement (CH50), and elevated soluble C5b‐9 represent the most severe anemia among the three reported patients. Despite this laboratory severity, he remained hemodynamically stable with preserved renal function, never requiring vasoactive support or dialysis, and recovered with warmed transfusion and thermal protection after eculizumab was deferred. Our experience suggests that organ failure, rather than anemia or hemolysis severity alone, may be a more clinically meaningful indicator for escalation to complement inhibition.

## 1. Introduction

Paroxysmal cold hemoglobinuria (PCH) is an acquired hemolytic anemia mediated by a biphasic anti‐P immunoglobulin G (IgG) antibody that binds red cells in the cold and fixes complement on rewarming, producing intravascular hemolysis. It is one of the more common causes of acute autoimmune hemolytic anemia in young children and typically follows a viral illness with symptom resolution over 1 to 2 weeks [1, 2]. Treatment is supportive, with thermal protection and warmed transfusion; corticosteroids have not been shown to shorten the course [2, 3]. For severe disease, two pediatric patients have received the terminal complement C5 inhibitor eculizumab, and a recent adult case received the classical pathway C1s inhibitor sutimlimab, so complement‐directed therapy is drawing growing interest [4–6]. No controlled trials or criteria define when a child requires treatment beyond supportive care. We report a child at the severe end of the hemolysis spectrum who recovered without complement inhibition or plasma exchange, and we compare him with the only two pediatric patients reported to have received eculizumab to ask where the threshold for complement inhibition should lie.

## 2. Case Description

A previously healthy 23‐month‐old male presented after approximately 1 week of viral upper respiratory symptoms with progressive pallor, scleral icterus, dark urine, and vomiting. He was tachycardic and ill‐appearing. Laboratory evaluation revealed a hemoglobin nadir of 2.7 g/dL, well below the median nadir of 5.5 g/dL reported across 230 cases in the largest systematic review [1], a leukemoid leukocytosis approaching 90 × 10^9^/L with preserved platelets (nadir 205 × 10^9^/L), acute kidney injury (AKI) with a creatinine near 1.9 mg/dL, lactate dehydrogenase (LDH) above the assay ceiling of 2500 U/L, indirect‐predominant hyperbilirubinemia, and 3+ hemoglobinuria. The degree of leukocytosis initially raised concern for leukemia, though the preserved platelet count argued against marrow infiltration. The reticulocyte response was increased, but inadequate for the degree of anemia, with an absolute reticulocyte nadir of 11 × 10^9^/L (reference range: 37–145 × 10^9^/L). The direct antiglobulin test (DAT) was polyspecific positive with weak IgG reactivity. Total hemolytic complement (CH50) was undetectable (reference range: 38.7–89.9 U/mL), and soluble C5b‐9 (sC5b‐9) was elevated at 427 ng/mL (reference range: < 250 ng/mL), consistent with brisk complement activation. The peripheral blood smear (PBS) showed spherocytes and leukoerythroblastic picture; no circulating blasts were identified. Preserved platelets and normal ADAMTS13 activity argued against thrombotic microangiopathy, and the PBS findings and subsequent course did not support leukemia.

He was admitted to the intensive care unit for severe DAT‐positive intravascular hemolysis and was initially managed with corticosteroids and red blood cell (RBC) transfusions. Methylprednisolone was escalated from 1 mg/kg every 8 h to pulse dosing of 30 mg/kg per day for 3 days (HD1‐3), then tapered and discontinued by HD9. Recurrent hemoglobin decline despite corticosteroids and transfusion support raised concern for a cold/complement‐mediated hemolytic process, prompting additional testing for cold autoantibodies and Donath–Landsteiner testing and a shift toward warmed supportive care. Warmed blood products and fluids were recommended when PCH was suspected and were added to nursing orders by HD5.

Between HD1‐6, he received 12 RBC transfusions in 5 and 10 mL/kg aliquots, totaling approximately 97 mL/kg (about 1550 mL at 16 kg). Throughout the course, he required no vasoactive support, had no lactic acidosis, sustained adequate urine output, and never required dialysis. Eculizumab was discussed on HD4‐5 given the severity of complement‐mediated hemolysis and the elevated sC5b‐9. The team documented explicit criteria for initiating complement inhibition, namely, inability to support hemoglobin levels with transfusion or new cardiovascular instability, and prepared a contingency plan of meningococcal vaccination, and antibiotic prophylaxis with a single 600 mg eculizumab dose if either developed.

**FIGURE 1:**
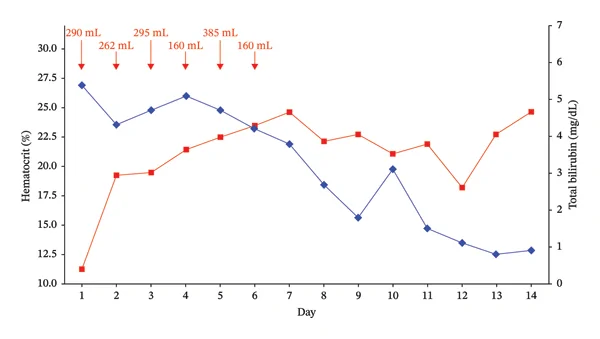
Trends in hematocrit and total bilirubin during hospitalization with transfusion support. Hematocrit (red squares, left *y*‐axis) and total bilirubin (blue diamonds, right *y*‐axis) were monitored over a 14‐day period. Red arrows indicate packed red blood cell transfusions, with the corresponding transfused volume (mL) shown above each arrow. Multiple transfusions were administered during HD1‐6, with cumulative daily transfusion volumes ranging from 160 to 385 mL. For visualization purposes, the curves are displayed continuously across HD5‐6, although hematocrit and bilirubin measurements were not obtained on those days. Therefore, the values shown between HD4‐7 represent graphical interpolation and should not be interpreted as directly measured laboratory results. At presentation, the patient exhibited severe anemia, with a hematocrit of approximately 11%, accompanied by marked hyperbilirubinemia (total bilirubin ∼5.4 mg/dL), consistent with active hemolysis. Following transfusion support during the first week of hospitalization, hematocrit increased progressively, reaching approximately 25% by HD7. Concurrently, total bilirubin demonstrated an overall downward trend, declining from greater than 5 mg/dL to less than 1 mg/dL by the end of the observation period. A transient increase in bilirubin was observed around HD10, followed by continued improvement. The inverse relationship between rising hematocrit and declining bilirubin is consistent with reduced hemolytic activity and restoration of red cell mass following transfusion therapy and clinical management. The sustained decline in bilirubin despite cessation of transfusions after HD6 suggests ongoing control of hemolysis beyond the immediate effect of red blood cell replacement. HD, hospital day.

Eculizumab was deferred because the hemoglobin remained supportable with transfusion, the creatinine had improved from 1.9 to 0.83 mg/dL by HD4 without complement blockade, and the absolute reticulocyte count had begun to rise by HD3, indicating early marrow recovery. The option of eculizumab and its associated risk of meningococcal infection were discussed with the family, who agreed with continued supportive care given the expected self‐limited course. Transfusion was no longer required after HD6, and hemolytic markers normalized over the following week (Figure 1). Sendout testing showed a weakly positive cold agglutinin screen with a cold agglutinin titer of 2, and positive Donath–Landsteiner, confirming the PCH diagnosis on HD10. *Klebsiella pneumoniae* bacteremia was also identified on HD10 and treated without altering the hemolytic course. He was discharged on HD15 with a hemoglobin of 7.6 g/dL and remained well at follow‐up. After Donath–Landsteiner antibodies were confirmed, transfusion medicine reinforced strict thermal protection as mainstay of therapy.

## 3. Discussion

PCH usually resolves within 1 to 2 weeks on supportive care alone [1, 2, 7]. To our knowledge, only two pediatric patients with PCH have received eculizumab, each as a single case report (Table 1). One developed shock and received eculizumab after corticosteroids failed [4]. The other developed sepsis with disseminated intravascular coagulation and dialysis‐dependent AKI and received plasma exchange followed by eculizumab [5]. In both, complement inhibition was prompted by organ failure rather than the degree of anemia or complement consumption, and both had a higher hemoglobin nadir (3.0 and 5.3 g/dL) than our patient (2.7 g/dL).

**TABLE 1:** Comparison of the present case with the two published pediatric cases of severe paroxysmal cold hemoglobinuria treated with eculizumab.

Feature	Lau–Braunhut 2019	Pelletier 2022	Present case
*Presentation*			
Age and sex	4 years, male	4 years, female	23 months, male
Antecedent trigger	Viral illness	Viral illness and recent vaccination	Viral upper respiratory illness

*Hemolysis and complement*			
Hemoglobin nadir (g/dL)	3.0	5.3	2.7
DAT pattern	C3 positive, IgG negative	C3 positive, IgG negative	Polyspecific positive, weak IgG
Complement studies	Baseline complement activation not reported	Complement‐consuming process; C4 normalized by HD14, values not reported	CH50 undetectable (< 12.5 U/mL); sC5b‐9427 ng/mL

*End-organ dysfunction*			
Shock or hemodynamic instability	Yes, shock (altered mental status, lactic acidosis, high oxygen extraction)	No, fluid‐responsive hypotension	No
Dialysis‐requiring AKI	No	Yes (CRRT then iHD)	No, AKI without dialysis

*Management and outcome*			
Corticosteroids	Methylprednisolone, not associated with clinical improvement	Given with eculizumab (refractory to supportive care)	Methylprednisolone, escalated to 30 mg/kg/day for 3 days, no clear effect
Therapy beyond supportive care	Eculizumab 600 mg once (HD4)	Plasma exchange (HD9); Eculizumab 600 mg once (HD15)	None
Red cell transfusion	Required before, none after eculizumab	11 transfusions, none after HD10	12 transfusions (∼97 mL/kg), HD1‐6
Outcome	Recovery; hemolysis resolved after eculizumab	Recovery with renal recovery, discharged HD24	Recovery, discharged HD15

*Note:* CH50, total hemolytic complement. iHD, intermittent hemodialysis.

Abbreviations: AKI, acute kidney injury; CRRT, continuous renal replacement therapy; DAT, direct antiglobulin test; HD, hospital day; sC5b‐9, soluble C5b‐9.

Our patient was the most anemic of the three, with undetectable CH50 and elevated terminal complement; however, he did not receive eculizumab, and yet he recovered on a timeline comparable to the treated cases. This pattern suggests that the degree of anemia and complement activation alone may not identify which patients require complement inhibition, and that hemodynamic instability and dialysis‐requiring organ injury may be more clinically meaningful indicators for its use.

These observations are hypothesis‐generating rather than practice‐changing. However, PCH is rare enough that controlled evidence may never exist, and clinicians facing severe disease must weigh the use of complement inhibition against the few reported cases. We, therefore, offer this comparison as provisional guidance rather than a validated threshold. In our experience, a child who is hemodynamically and renally stable may be observed with thermal protection and warmed transfusion, even with profound anemia and undetectable complement, while the reticulocyte count recovers. Clinicians may consider complement inhibition when a child requires the kind of intensive support seen in the two treated patients, namely, vasoactive support for shock or dialysis for kidney injury, or when persistent reticulocytopenia accompanies refractory hemolysis.

The complement consumption itself was interpreted as a consequence of the biphasic hemolysin rather than as evidence of an independent complement‐driven process such as atypical hemolytic uremic syndrome, and improving renal function without blockade supported this interpretation. Deferring eculizumab also avoided the recognized risk of meningococcal infection with terminal complement inhibition, the meningococcal vaccination, and antibiotic prophylaxis it would have required in a 23‐month‐old, and the substantial drug cost.

This case also illustrates that PCH may present with an IgG‐positive DAT, which is atypical and should not deter suspicion of a cold‐mediated process, especially when the corticosteroid response is poor. Valid Donath–Landsteiner testing depends on careful specimen handling. Blood must be drawn into warmed tubes and allowed to clot in an upright position at body temperature, which the blood bank achieves with a plasma thawer. The Donath–Landsteiner sample is a plain red‐top clot tube, and at least 4 mL of serum, not whole blood, is required. Without these precautions, the test may be falsely negative or invalid, and the diagnosis delayed.

This comparison is limited by its evidence base of two single case reports, which cannot establish a threshold, and by publication bias, because treated patients are reported more often than those who recover untreated, skewing the published literature toward higher acuity. Both treated cases are also confounded, since Pelletier’s patient received plasma exchange, dialysis, and eculizumab together and Lau–Braunhut’s received eculizumab with thermal protection, so recovery cannot be attributed to the drug alone. Because PCH usually self‐resolves, supportive care is the default and the bar for an unproven therapy is high, and improvement on a self‐limiting disease is hard to credit to eculizumab, which has itself failed in one report [8]. Our case cannot prove complement inhibition is never needed at this severity, nor can it establish that anemia severity should not influence treatment decisions. It shows only that one child is more anemic than either treated patient, with undetectable complement, recovered on supportive care.

As complement inhibitors are used more often without a guiding standard, defining when a stable but severely anemic child with PCH benefits from eculizumab will be important. Given the rarity of PCH, settling this will require a prospective trial or a multicenter retrospective study.

## 4. Ethical Approval

In accordance with UCSF Human Research Protection Program guidance, this single‐patient clinical case report does not constitute human subjects research and did not require Institutional Review Board review. Written informed consent for publication of this deidentified report was obtained from the patient’s legal guardian. Additionally, this study was not designed as a clinical trial and therefore does not have a clinical trial registration number.

**Nomenclature**
AKI: Acute kidney injury
CH50: Total hemolytic complement
DAT: Direct antiglobulin test
IgG: Immunoglobulin G
HD: Hospital day
LDH: Lactate dehydrogenase
PBS: Peripheral blood smear
PCH: Paroxysmal cold hemoglobinuria
RBC: Red blood cell
sC5b‐9: Soluble C5b‐9 (terminal complement complex)

## Funding

No funding was received.

## Conflicts of Interest

The authors declare no conflicts of interest.

## Supporting Information

Additional supporting information can be found online in the Supporting Information section.

## Supporting information


**Supporting Information** CARE‐checklist‐English‐2013.

## Data Availability

Data sharing is not applicable to this article as no new datasets were generated or analyzed during the current study.

## References

[1] Jacobs J. W. , Figueroa Villalba C. A. , Booth G. S. , Woo J. S. , Stephens L. D. , and Adkins B. D. , Clinical and Epidemiological Features of Paroxysmal Cold Hemoglobinuria: A Systematic Review, Blood Advances. (2023) 7, no. 11, 2520–2527, 10.1182/bloodadvances.2022009516. 36716137 PMC10248093

[2] Cooling L. L. , Kids, Colds, and Complement: Paroxysmal Cold Hemoglobinuria, Transfusion. (2017) 57, no. 6, 1332–1335, 10.1111/trf.14128. 28594143

[3] Voulgaridou A. and Kalfa T. A. , Autoimmune Hemolytic Anemia in the Pediatric Setting, Journal of Clinical Medicine. (2021) 10, no. 2, 10.3390/jcm10020216. PMC7828053 33435309

[4] Lau-Braunhut S. A. , Stone H. , Collins G. , Berentsen S. , Braun B. S. , and Zinter M. S. , Paroxysmal Cold Hemoglobinuria Successfully Treated With Complement Inhibition, Blood Advances. (2019) 3, no. 22, 3575–3578, 10.1182/bloodadvances.2019000897. 31738828 PMC6880888

[5] Pelletier J. , Ward C. , Borloz M. , Ickes A. , Guelich S. , and Edwards Jr. E. , A Case of Childhood Severe Paroxysmal Cold Hemoglobinuria With Acute Renal Failure Successfully Treated With Plasma Exchange and Eculizumab, Case Reports in Pediatrics. (2022) 2022, no. 1, 3267189–5, 10.1155/2022/3267189. 35497647 PMC9054456

[6] Sato K. , Nakamura Y. , Hara R. et al., Adult-Onset Severe Paroxysmal Cold Hemoglobinuria After COVID-19 Successfully Treated With Sutimlimab, International Journal of Hematology. (2024) 120, no. 6, 735–742, 10.1007/s12185-024-03851-8. 39304597

[7] Jacobs J. W. , Booth G. S. , Woo J. S. , Stephens L. D. , Figueroa Villalba C. A. , and Adkins B. D. , Challenges in Recognising Paroxysmal Cold Hemoglobinuria, Transfusion Medicine. (2024) 34, no. 1, 71–73, 10.1111/tme.13024. 38151804

[8] Gregory G. P. , Opat S. , Quach H. , Shortt J. , and Tran H. , Failure of Eculizumab to Correct Paroxysmal Cold Hemoglobinuria, Annals of Hematology. (2011) 90, no. 8, 989–990, 10.1007/s00277-010-1123-x. 21110191

